# Anti-oomycete activities from essential oils and their major compounds on *Phytophthora infestans*

**DOI:** 10.1007/s11356-023-29270-6

**Published:** 2023-10-02

**Authors:** Caroline Deweer, Karin Sahmer, Jérôme Muchembled

**Affiliations:** 1 Joint Research Unit 1158 BioEcoAgro, INRAE, JUNIA, University of Lille, University of Liège, UPJV, University of Artois, ULCO, F-59000 Lille, France; 2 Univ. Lille, IMT Lille Douai, Univ. Artois, JUNIA, ULR 4515 - LGCgE, Laboratoire de Génie Civil et géo-Environnement, F-59000, Lille, France

**Keywords:** Botanicals, Essential oils, Biofungicides, *Phytophthora infestans*, IC_50_, Anti-oomycete activity

## Abstract

Botanicals are various plant-based products like plant extracts or essential oils. Anti-fungal activities of selected essential oils were tested on the pathogen causing potato and tomato late blight (*Phytophthora infestans*). Tests to evaluate anti-oomycete activities of commercial essential oils and their major compounds were carried out in vitro in microplate in liquid media. Anti-oomycete activities on *Phytophthora infestans* strain were obtained from essential oils/major compounds: *Eucalyptus citriodora*/citronellal; *Syzygium aromaticum* (clove)/eugenol; *Mentha spicata*/D-Carvone, L-Carvone; *Origanum compactum*/carvacrol; *Satureja montana* (savory)/carvacrol; *Melaleuca alternifolia* (tea tree)/terpinen-4-ol, and *Thymus vulgaris*/thymol. As an active substance of mineral origin, copper sulfate was chosen as a control. All selected essential oils showed an anti-oomycete activity calculated with IC_50_ indicator. The essential oils of clove, savory, and thyme showed the best anti-oomycete activities similar to copper sulfate, while oregano, eucalyptus, mint, and tea tree essential oils exhibited significantly weaker activities than copper sulfate. Clove essential oil showed the best activity (IC_50_ = 28 mg/L), while tea tree essential oil showed the worst activity (IC_50_ = 476 mg/L). For major compounds, three results were obtained: they were statistically more active than their essential oils (carvacrol for oregano, D- and L-Carvone for mint) or as active as their essential oils sources (thymol for thyme, carvacrol for savory, terpinen-4-ol for tea tree) or less active than their original essential oils (eugenol for clove, citronellal for eucalyptus). Microscopical observations carried out with the seven essential oils showed that they were all responsible for a modification of the morphology of the mycelium. The results demonstrated that various essential oils show different anti-oomycete activities, sometimes related to a major compound and sometimes unrelated, indicating that other compounds must play a role in total anti-oomycete activity.

## Introduction

In agriculture and plant protection, botanicals are plant-based products having potentially less negative impact on non-target organisms and less environmental risks than classical pesticides (Regnault-Roger and Philogène 2008; Alonso-Gato et al. 2021). In conventional agriculture, botanicals could be interesting to reduce the use of chemical pesticides causing chronic pollution (Nicolopoulou-Stamati et al. 2016). In organic farming, botanicals could be interesting to reduce the use of copper-based products: due to its ability to accumulate in soils without being degradable and its ability to bioaccumulate in organisms, copper can also cause environmental pollution (EFSA 2018; La Torre et al. 2018; Gajewska et al. 2022). Botanicals could be an alternative to chemical and mineral pesticides especially since they have been considered as ecochemical products for the sustainable management of crop pests and diseases aiming to improve the safety of the producer, consumer, and the environment (Cavoski et al. 2011; Campos et al. 2019; Drakopoulos et al. 2020). Essential oils (EOs) are considered as botanicals. EOs are the product of secondary metabolism in plants. As specialized metabolites, they have several roles such as attracting pollinating insects or repelling certain pests. EOs can also act as appetizers or repellents or as growth disrupters for phytophagous insects (Bakkali et al. 2008; Isman et al. 2011). The specialized metabolites of plants have evolved to protect them from attack by microbial pathogens (Benner 1993). Biological properties of EOs are well-known since a long time. EOs have antiseptic, medicinal, and perfume properties. They are used, sometimes for centuries, for embalming, food preservation, as analgesic, sedative, anti-inflammatory, spasmolytic, and local anesthetic insects (Bakkali et al. 2008).

EOs are natural liquids produced generally by hydrodistillation. They can be extracted from all plant organs: buds, flowers, stems, seeds, fruits, roots, wood (Jugreet et al. 2020). EOs are composed of many compounds, generally, around 20–60 components with different concentrations; some are major components at fairly high concentrations (20–70%), while others are present in trace amounts (Bilia et al. 2014; Pavela and Benelli 2016). The composition of EOs can be variable according to the growth conditions of the plant from which they are extracted (abiotic and biotic stress) (Seow et al. 2014). From the chemical point of view, EOs represent a complex and unique mixture of compounds, specific for each plant and extraction procedure, including, but not limited to alkaloids, flavonoids, isoflavones, monoterpenes, phenolic acids, and aldehydes (Seow et al. 2014).

In literature, a lot of laboratory work was carried out on the use of EOs in plant protection to manage weeds (herbicidal effect), insects (acaricidal, nematicidal, and insecticidal effect), and causal agents of diseases (virucidal, bactericidal, and fungicidal effect), and there are many review articles that summarize it (Bakkali et al. 2008; Jugreet et al. 2020; Pavela and Benelli 2016; Kalemba and Kunicka 2003; Yoon et al. 2013 ; Nazzaro et al. 2017; Campolo et al. 2018; Fierascu et al. 2020; Ikbal and Pavela 2019 ; Ibáñez and Blázquez 2020 ; Raveau et al. 2020). Regarding the anti-fungal properties of EOs, distinctions were made between toxigenic (Dambolena et al. 2012), post-harvest (Lee et al. 2007; Combrinck et al. 2011; Camele et al. 2012; Shao et al. 2013), or phytopathogenic fungi (Terzi et al. 2007; Dan et al. 2010; Kadoglidou et al. 2011; Riccioni and Orzali 2011; Sharma et al. 2017). Concerning oomycetes, very few studies were carried out compared to fungi. On essential oils as potential alternative biocontrol products, only 14 articles about oomycetes on a total of 155 concerning anti-fungal activities in the last decade (less than 10%) were mentioned (Raveau et al. 2020). Potato late blight is caused by an oomycete, *Phytophthora infestans*, one of the most devastating plant pathogens, infecting many species in the Solanaceae family, particularly potato and tomato. According to Andrivon (1996), the microorganism would be originated in Central or South America and would have been imported into the USA around 1842. From the USA, the disease spread to Europe, with the first description of the disease in Belgium and the Netherlands in 1845. The vector for the spread of the disease probably consisted of tubers intended for seed which were infected. It was potato late blight that caused the great potato blight in Ireland from 1845 to 1851, killing millions of people (Andrivon 1996) and continues to appear in new locations and new intensity (Fry et al. 2015). *Phytophthora infestans* is responsible for an estimated US$ 5 billion of damage each year (Judelson and Blanco 2005). Because of the evolution of resistance in pest populations and to ensure the sustainability of agriculture, research and development of new control methods are necessary (Chandler et al. 2011; Barzman et al. 2015; Czaja et al. 2015; Lamichhane 2017).

Among the new integrated pest management, the use of specialized metabolites from plants could be a way to explore. There are many more studies showing that a range of botanical compounds could be used (Borges et al. 2018). For example, molecules from different plant extracts inhibited the disease development of *P. infestans* (Kim et al. 2004; Goufo et al. 2008; Shim et al. 2009; Moushib et al. 2013; Švecová et al. 2017; Mugao et al. 2020). EO from S*almea scandens* showed an anti-fungal activity against *Phytophthora infestans* (mycelial radial growth and MIC) (Villa-Ruano et al. 2015). Soylu et al. (2006) tested the contact and volatile effect of EOs (thyme, oregano, rosemary, lavender, fennel, and laurel) in solid media (radial mycelial growth). And Jacquin et al. (2022) showed a very weak anti-oomycete activity from hop cone EO in liquid medium. So, the aim of this article is to test selected EOs (some EOs never tested on *P. infestans* in liquid medium as *Satureja montana, Eucalyptus citriodora, Mentha spicata*) and their major compounds on spore germination in a liquid medium (sealed microplates) to calculate the IC_50_ and the anti-oomycete activities. EOs were selected by technical experts in organic farming.

## Materials and methods

### Fungal isolate and inoculum production for in vitro assays

The culture of *Phytophthora infestans* (MUCL ref 54355) was done on agar medium V8, without light and at 18 °C. A filtered suspension of 4 × 10^4^ sporangia/ml was carried out for the performance of microplate assays in glucose peptone medium from 20-day-old cultures. The sporangia suspension was placed for 1 h at 4 °C before beginning the microplate assays.

### Essential oils, major compounds, and copper sulfate

Eucalyptus (*Eucalyptus citriodora*, Golgemma, lot n°B394N017), clove (*Syzygium aromaticum*, Golgemma, lot n°B481N005), mint (*Mentha spicata*, SAPAD, lot n°02BC1217813BN), oregano (*Origanum compactum*, Golgemma, lot n°B857007), savory (*Satureja montana*, Golgemma, lot n°B857007), tea tree (*Melaleuca alternifolia*, Golgemma, lot n° B891N009), and thyme (*Thymus vulgaris*, Golgemma, lot n°B924N0114) were the seven selected EO used for experiments.

**Fig. 1:**
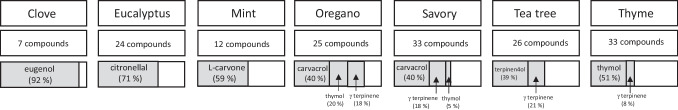
The seven selected commercial EOs: the total number of compounds quantified and the relative percentage of major compound tested per EO (according to Muchembled et al. 2018)

We already characterized these EOs by GC-MS in a previous study. Their anti-fungal activities had been evaluated in vitro on *Venturia inaequalis* spores (Muchembled et al. 2018). Figure 1 shows the total number of compounds and reminds the relative proportion of the majority compound present per EO tested.

The major compounds of each EO were tested to assess the importance of their fungicidal activities compared to those of the EOs. Carvacrol (Sigma-Aldrich, W224511), D-Carvone (Sigma-Aldrich, 22020), L-Carvone (Sigma-Aldrich, W224901), citronellal (Sigma-Aldrich, 373753), eugenol (Sigma-Aldrich, E51791), terpinen-4-ol (Sigma-Aldrich 86477), γ-terpinene (Sigma-Aldrich, 86476), and thymol (Sigma-Aldrich, T0501) are the most relevant compounds (Muchembled et al. 2018) and were purchased to carry out the test. Copper sulfate (Merck, 92HF136288) was chosen as mineral active substance.

### Anti-oomycete activity

EOs and major compounds were tested in a liquid medium in microplates. A range of concentrations (Table 1) was made for each product with glucose peptone (14.3 g of glucose and 7.1 g bactopeptone per liter) according to Muchembled et al. (2018).

**Table 1:** Concentrations range for products used for in vitro test

Tested products	Unit	Concentrations							
Essential oils	vol/vol %	0	0.0002	0.0008	0.0031	0.0125	0.05	0.2	0.8
Major compounds	mg/L	0	0.98	3.91	15.63	62.5	250	1000	4000
Mineral fungicides	mg/L	0	0.11	0.4	1.4	4.9	17.14	60	120

**Fig. 2:**
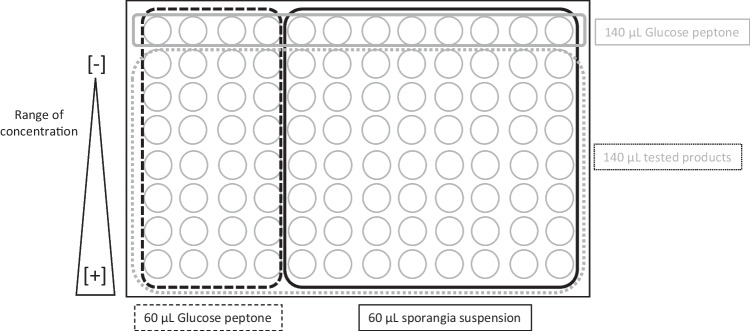
Test layout in microplate for each product

A total of 140 μL/well were distributed into 96-well microplates, one concentration per line, composed of 8 wells with spore suspension and 4 without (Fig. 2). The first 8 wells were filled with 60 μL of a spore suspension, and the 4 wells without spore were used as control to measure the net optical density (OD).

The plates were then sealed and stirred (140 rpm) at 20 °C for 6 days. The optical density (OD) was read at 635 nm with a microplate reader. Net OD obtained depending on concentrations were used to calculate the IC_50_ (inhibiting concentration 50%) using a non-linear regression according to Muchembled et al. (2018). The experiments were performed at least three times independently to estimate the variability intra and inter experiments. An *F* test with an *α* risk of 5% was used in order to test if there were any differences between modalities. If this was the case, a pairwise comparison of IC_50_ values of EOs and major compounds was performed based on confidence intervals with Bonferroni adjustment. These statistical analyses were done with R software (R Core Team. 2019) and its nlstools package (Baty et al. 2015).

### Morphological observations

After 6 days of incubation, microscopic observations were made. Samples of 5 μL were taken in wells with a concentration near to the IC_50_ and were compared to untreated control. Five microliter of lactophenol blue was added to samples to be observed under an optical microscope in order to check the fungus morphology (Nikon Eclipse 80i, with Nikon Digital Camera Ddxm1200c).

## Results

### Biological results

All selected EOs showed an anti-oomycete activity materialized by an IC_50_ (Table 2). With an IC_50_ of 28.42 mg/L, clove EO had the smallest IC_50_, hence, the largest activity. The other EOs are as follows (in increasing order of the IC_50_): savory, oregano, thyme, eucalyptus, mint, and tea tree. Table 2 also shows confidence intervals for the IC_50_s, allowing to see if the observed differences are statistically significant.

**Table 2:** IC_50_ of EOs, major compounds, and copper sulfate on *Phytophthora infestans* with Bonferroni adjusted confidence intervals with a global confidence of 95% (three independent experiments)

	IC_50_ inf (mg/L)	IC_50_ (mg/L)	IC_50_ sup (mg/L)
Clove	9.16	28.42	58.43
Eugenol	79.85	125.69	156.24
Eucalyptus	61.4	122.11	276.37
Citronellal	337.3	840.7	1016.07
Mint	116.52	130.56	176.74
D-Carvone	66.68	89.84	114.64
L-Carvone	65.99	76.08	113.79
Oregano	76.64	96.5	111.41
Savory	43.61	74.65	90.43
Carvacrol	25.51	53.72	60.11
Tea tree	353.4	476.37	568.84
Terpinen-4-ol	267.94	315.8	407.44
g-terpinene	NC	NC	NC
Thyme	39.48	99.41	128.88
Thymol	71.99	96.24	130.97
Copper sulfate	15.76	19.71	51.63

All major compounds showed an anti-oomycete activity too except for γ-terpinene whose IC_50_ could not be calculable because spore germination was not inhibited. With an IC_50_ of 125.69 mg/L, the activity of eugenol appeared far from clove EO activity even if eugenol represents 92% of its composition. For the least active EO, terpinen-4-ol showed an IC_50_ quite close to the activity of tea tree EO while terpinen-4-ol represents only 39% of the composition. Citronellal from eucalyptus EO showed the same type of result as eugenol from clove EO: with an IC_50_ of 840.7 mg/L, the activity of citronellal appeared far from eucalyptus EO activity (122.11 mg/L) even if citronellal represents 71% of its relative composition. Carvacrol from savory EO (40%) and thymol from thyme EO (51%) showed similar results in comparison with terpinen-4-ol and tea tree EO: the activity of carvacrol (53.72 mg/L) was close to savory EO (74.65 mg/L), and the activity of thymol (96.24 mg/L) was similar to thyme EO (99.41 mg/L). We observed different results with carvacrol from oregano EO (40%) and carvone from mint EO (59%). Carvacrol showed better activity than oregano EO (53.72 mg/L against 96.5 mg/L respectively). Likewise, D- and L-Carvone showed better activity (89.84 mg/L and 76.08 mg/L respectively) than mint EO (130.56 mg/L).

**Fig. 3:**
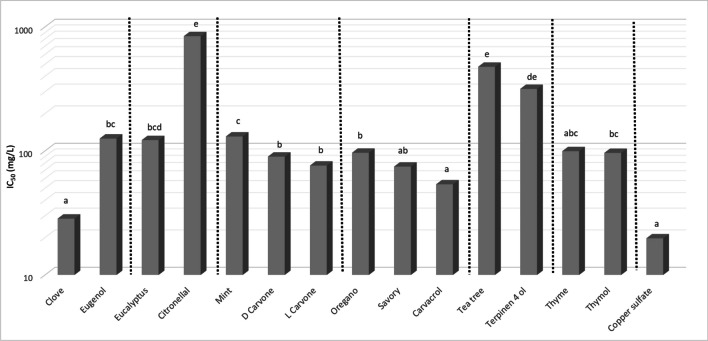
Anti-oomycete activities of essential oils, major compounds, and copper sulfate on *Phytophthora infestans*

We observed in Fig. 3 that the activity of clove EO was not statistically different from savory and thyme. Similarly, the anti-oomycete activities of savory and oregano EO were not statistically different for very identical compositions (40% carvacrol and 18% γ-terpinene in the two cases). These EOs also showed no statistically significant difference to thyme and eucalyptus EOs. For the differences in activity between the EOs, statistical analysis demonstrated that (i) clove EO was more active than oregano and mint EO, (ii) oregano EO was more active than mint EO, and (iii) tea tree EO was less active than all the other EOs.

Concerning the comparison of the activities of the majority compounds with their original EOs, the statistical analysis showed three trends. Firstly, some major compounds presented the same level of the activity as EOs (carvacrol for savory EO, thymol for thyme EO, and terpinen-4-ol for tea tree EO). Secondly, some major compounds presented a better activity than their original EOs (carvacrol for oregano EO, D- and L-Carvone for mint EO with no isomeric effect between D- and L-Carvone). Thirdly, other major compounds presented a less important activity than EOs (eugenol for clove EO and citronellal for eucalyptus EO). Finally, the activity of clove, thyme, and savory EOs as well as the activities of carvacrol were the same as copper sulfate.

### Microscopic observations

**Fig. 4:**
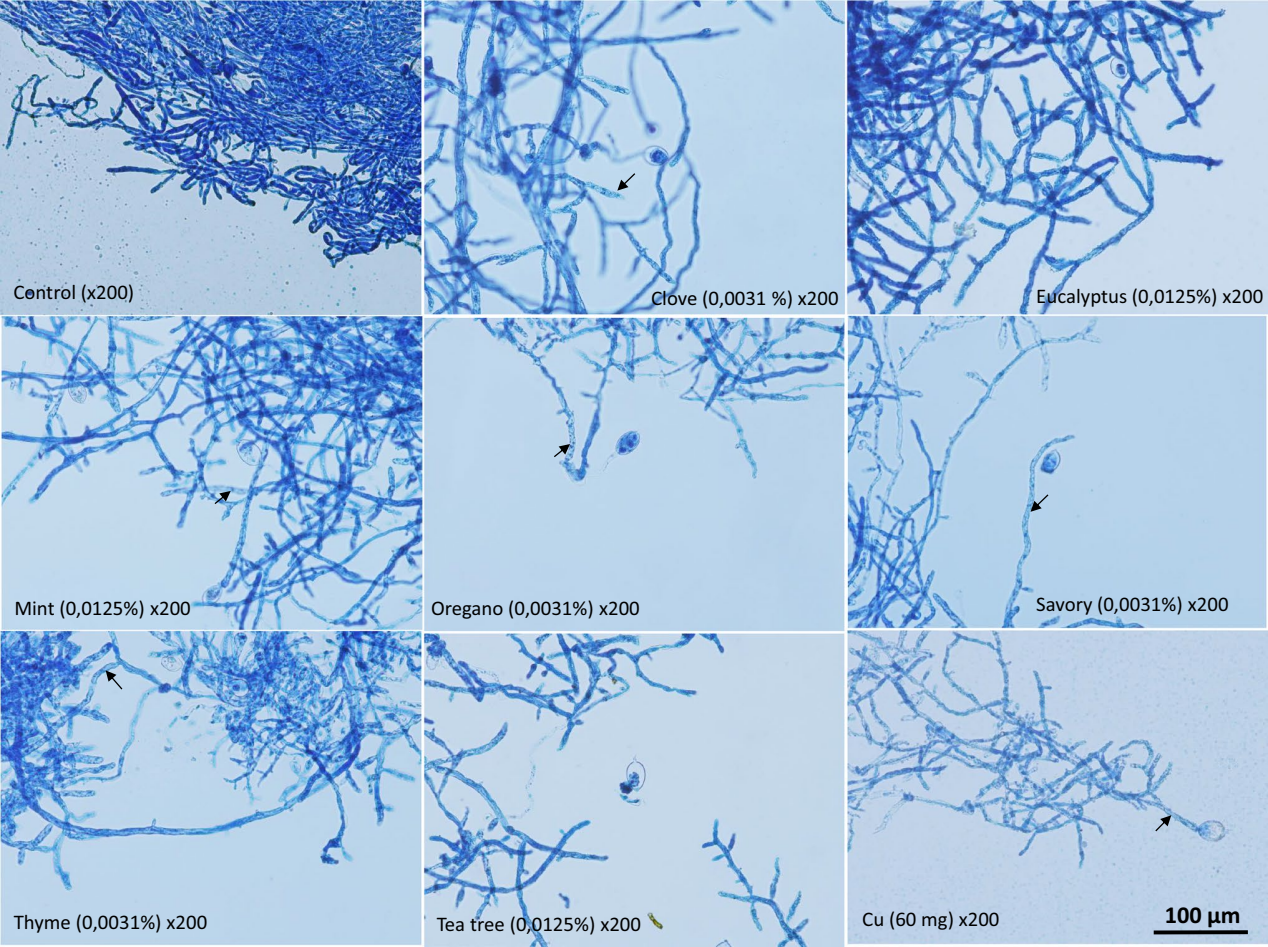
Effect of EOs and copper sulfate on the morphology of *Phytophthora infestans* mycelium. Arrows indicate cytoplasmic coagulation in the mycelium

Microscopic observations showed differences between the control, EOs, and copper sulfate (Fig. 4). The mycelium of the control was intact and dense. The hyphal morphology showed degenerative changes when *P. infestans* mycelium was in contact with EOs especially for clove, oregano, savory, and thyme. The fungus continued to grow but the mycelium seemed to be affected by the different products. A cytoplasmic coagulation was observed (arrows) in agreement with previous work (Soylu et al. 2006). The hyphae appeared degraded when the fungus was exposed to copper sulfate too.

## Discussion

We demonstrated that the EOs of clove, savory, and thyme had the best anti-oomycete activity, while tea tree EO had the least one.

In the current study, the IC_50_ of clove EO was 28.42 mg/L (9.16–58.43). Compared to studies conducted over the past decades, clove EO in solid media strongly inhibited mycelial growth at the lowest concentration of 0.41 μL/mL and showed an ED_50_ of 5 μL/mL on sporangial germination of *P. infestans* (Najdabbasi et al. 2020). Nana et al. (2015) estimated the MIC of clove EO on *Phytophthora megakarya* at 250 μL/L on solid medium. For *P. parasitica*, the highest anti-oomycete activity was highlighted for clove EO with until 100% inhibition and 3.6% for ED_50_ with disc diffusion method in solid media (Diánez et al. 2018). Although methods of test were different, clove EO always shows regular anti-oomycete activities.

The IC_50_ of EO from *Satureja montana* was 74.65 mg/L (43.61–90.43) under our experimental conditions. There are some rare articles on the anti-fungal activity of savory, but none with *Satureja montana*. *S. hortensis*, *S. spicigera,* and *S. khuzistanica* EOs showed an important anti-fungal activity on mycelial growth of *Penicillium digitatum*, *Botrytis cinerea*, and *Rhizopus stolonifera* (Farzaneh et al. 2015). *Satureja khuzistanica* EO was also the most effective in inhibiting mycelial growth from 100 μL/L on *P. palmivora* (Sarkhosh et al. 2018). In these two studies, the authors revealed that the significant anti-fungal activity seemed to be linked to the presence of carvacrol and γ-terpinene. This correlated well with the composition of the EO of *Satureja montana* that we used and could also explain the high activity we measured in liquid medium.

For oregano EO, we showed an anti-oomycete activity with an IC_50_ of 96.50 mg/L (76.64–111.41) which is similar to the IC_50_ of savory EO. This seems to be related to the same proportion of carvacrol and γ-terpinene measured in the composition of the two EOs. Published results showed that oregano EO to 100 ppm caused mycelial growth reduction of *P. infestans* on Rye B medium (Olanya and Larkin 2006). Similarly, a contact dose effect was shown with *Origanum syriacum* EO with carvacrol chemotype on V8 juice agar medium amended with antibiotics (Soylu et al. 2006). Oregano EO was therefore capable of inhibiting mycelium growth and sporangia germination regardless of the nature of the culture medium.

For thyme EO whose major compound is thymol, we highlighted an IC_50_ of 99.41 mg/L (39.48–128.88). Investigations in solid media using *T. vulgaris* EO with thymol chemotype showed a good activity for inhibiting mycelial growth of *P. infestans* (Quintanilla et al. 2002). However, no data about IC_50_ was available. In liquid media, an important inhibitory effect on sporangial germination was shown for *Thymus vulgaris* EO without any information about its composition (Najdabbasi et al. 2020). Consequently, the exact composition and chemotype defined in the current study are of interest for a better understanding of results from previous and further studies on *T. vulgaris* EO.

For *Eucalyptus citriodora* EO with citronellal chemotype, an anti-oomycete activity on *P. infestans* with an IC_50_ of 122.11 mg/L (61.40–276.37) was highlighted for the first time. For the anti-fungal activity, only few information on *Eucalyptus* EO with *citriodora* species are available. Conversely, the biological effects of *Eucalyptus globulus* EO whose main compound is eucalyptol are more described. For instance, Sharma et al. (2017) obtained an IC_50_ of 207 ppm for *Eucalyptus globulus* EO in solid medium on *Fusarium oxysporum.* This value is similar to ours even if the nature of the EOs, the methodology of the test, and the targeted organisms are different.

An anti-oomycete activity of mint EO with an IC_50_ of 130.56 mg/L (116.52–176.74) was measured in the current study. Once again, we are the first to produce an IC_50_ for *Mentha spicata* EO (carvone chemotype) on *P. infestans* in liquid medium. Most studies on oomycetes were indeed carried out with *Mentha piperata* EO (menthol chemotype) (Sarkhosh et al. 2018; Diánez et al. 2018) or with other species of *Mentha* (Clerck et al. 2020). In these studies, mint EO was never the most active EO, which was also observed here.

The weakest anti-oomycete activity with an IC_50_ of 476.37 mg/L (353.40–568.84) was highlighted for tea tree EO. This agrees with other data produced in solid medium which demonstrated that tea tree EO, whose composition characterized by GC-MS was similar to ours, was generally less effective than other EOs (Bishop and Thornton 1997; Riccioni and Orzali 2011; Sarkhosh et al. 2018).

Thanks to the IC_50_ calculation, we highlighted the differential activities of seven EOs selected by technical experts in organic farming (French CASDAR project). Usually, EOs had little or no activity in *P. infestans* since it was shown during a screening that only ten out of ninety EOs had activity (Clerck et al. 2020). The seven EOs have been well selected by the experts. They were therefore all more active that the EO of hop cones tested under strictly identical conditions by Jacquin et al. (2022) (IC_50_ of 5355 mg/L (1714–9775)). However, we showed that none was more effective than copper sulfate, but we also demonstrated that clove, savory, and thyme EOs were as active as copper sulfate.

In our experiments with major compounds, carvacrol (IC_50_ of 53.72 mg/L (25.51–60.11)) was as effective as savory EO and more effective than oregano EO. Carvacrol was also statistically more effective than thymol (IC_50_ = 96.24 mg/L (71.99–130.97)). Our results confirm the findings of Dambolena et al. (2012) who showed a greater activity of carvacrol compared to thymol and a greater activity of an EO with carvacrol chemotype compared to an EO with thymol chemotype (Ben Jabeur and Hamada 2015).

**Fig. 5:**
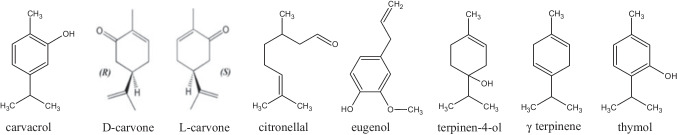
Chemical structure of main compounds of EOs

γ-Terpinene and terpinen-4-ol have almost the same molecular structure (Fig. 5), but they did not exhibit the same activity. It was impossible to calculate an IC_50_ for γ-terpinene indicating no anti-oomycete activity for this molecule, while for terpinen-4-ol (IC_50_ = 315.80 mg/L (267.94–407.44)), anti-oomycete activity was at the same level as tee tree EO. Previous work on *Fusarium* sp. showed also that terpinen-4-ol was responsible for the anti-fungal activity of the tee tree EO (Terzi et al. 2007). The difference of activity between terpinene-4-ol and γ-terpinene is based on the chemical function of terpinene-4-ol as an alcohol (Hammer et al. 2003; Kadoglidou et al. 2011). Our results were consistent with several studies indicating that terpenes like γ-terpinene are inefficient as antimicrobials and antifungals when they are applied as single compounds (Camele et al. 2012; Hyldgaard et al. 2012).

For eugenol, we showed an anti-oomycete activity with an IC_50_ of 125.69 mg/L (79.85–156.24) which is statistically different from the IC_50_ of clove EO. With 90% of the composition, it was curious to note that clove EO activity was not embodied by eugenol alone. Moreover, with the same experimental methodology, eugenol presented the same level of activity as clove EO on *Venturia inaequalis* (Muchembled et al. 2018). Eugenol interacts with plasma membranes and binds to certain proteins, but its main mode of action is membrane permeabilization, disrupting the electrochemical gradient between intra- and extracellular media, and causing the “leakage” of cell contents (Rhayour et al. 2003; Hyldgaard et al. 2012). The difference of sensibility for eugenol between the oomycete *P. infestans* and the fungus *V. inaequalis* is therefore certainly due to a difference in the nature of their membranes. D-Carvone and L-Carvone were more effective on *P. infestans* than mint EO in our experiments. With 60% of the total composition, carvone is typical of spearmint EO. Most studies indicated that carvone was responsible for spearmint EO activity but without information on isomeric forms and not on oomycetes (Kadoglidou et al. 2011; Benomari et al. 2018). The higher percentages of oxygenated compounds like (-)-carvone were shown to have stronger fungi growth inhibitions (Ben Salha et al. 2019). The comparison of carvone enantiomers was done on different pathogens in vitro and showed that the R-enantiomer inhibited all the pathogens at a lower concentration, except for *Alternaria citri*, towards which the S-enantiomer was more active (Combrinck et al. 2011). No difference between the two enantiomers’ activity was observed in the current study.

We showed for the first time that citronellal (monoterpenoid) was one of the least effective major compounds (IC_50_ = 840.70 mg/L (337–1016)) on *P. infestans*. Citronellal represents 70% of the *Eucalyptus citriodora* EO composition. However, citronellal activity was statistically lower than *E. citriodora* EO. It is possible that other compounds such as isopulegol may interact with citronellal, isopulegol being present in small quantities in eucalyptus EO and responsible for a strong anti-fungal activity (Montenegro et al. 2020). On fungi, citronellal also showed the weakest anti-fungal activity (Marei and Abdelgaleil 2018; Wang et al. 2018). Citronellal showed recently to act on membrane integrity and ergosterol synthesis in the fungus *Penicillium digitatum* (OuYang et al. 2021) and to disturb chitin synthesis in the fungus *Magnaporthe orizae* (Zhou et al. 2022). For the oomycete *Phytophthora infestans*, the nature of the membrane and cell wall are different from the classical fungi with no ergosterol in the membrane and no chitin in the cell wall (Lee et al. 2012; Wang et al. 2021). Citronellal could therefore not act exactly in oomycetes as in fungi. The in vitro evaluations of major compounds confirmed that the most notable anti-oomycete activity belonged to carvacrol, then to D-Carvone and L-Carvone, thymol, and eugenol. Besides, terpinen-4-ol and citronellal showed moderate to weak activity, while γ-terpinene was an inefficient compound on *P. infestans.* Carvacrol was the only compound to be as effective as copper sulfate.

Our experiments were carried out in vitro to evaluate the anti-oomycete activity of seven selected EOs and their major compounds on spore germination of *P. infestans*. If some EOs could have a future within the framework of integrated protection against potato late blight, it would be necessary to select EOs that are not only effective to protect plants against *P. infestans* but also without any phytotoxic effect on potato. Bainard et al. (2006) already showed indeed that the phytotoxicity of clove EO on broccoli leaves was 2.5% (0.8% of clove EO was the maximum for our manipulations). Moreover, Werrie et al. (2020) showed that the phytotoxicity depends on the application systems and mode of action according to the EOs and major compounds. Specific studies on the toxicity of EOs will be necessary in the near future. In the same way, it would be interesting to better know the mode of action of essential oils and their major compounds. Effectively, the specific mode of action of EOs on oomycetes is not yet known in detail contrary to fungi for which the mode of action of EOs is well referenced. EOs act at two levels in fungi. The first level is the fungal cell with inhibition of the cell wall formation and disruption of the cell membrane (importance of ergosterol). The second level is within the cell with the mitochondrial dysfunction and the efflux pumps inhibition (Nazzaro et al. 2017). That is well-known in fungal kingdom but is much less known in oomycetes (stramenopiles supergroup) such as *P. infestans* whose cell wall and membrane have different constitutions from classical fungi (Aronson et al. 1967; Lee et al. 2012; Wang et al. 2021). Due to their wide composition, the mode of action of EOs in oomycetes and fungi seem to be multiple. So, tea tree EO which at first classified by FRAC (Fungicide Resistance Action Committee) as a cell membrane disruptor (F7 FRAC code) was recently reclassified as BM01 (Biologicals with Multiple modes of action) for example.

## Conclusion

The use of sealed microplate allowed to effectively compare the anti-oomycete activity of several EOs, some of which were never tested on *Phytophthora infestans* (*Satureja montana, Eucalyptus citriodora, Mentha spicata* EOs). The use of sealed microplate in liquid medium presented several interests. The risk of volatility is weak (i) when the mixture was prepared in the wells as opposed to the mixture carried out in a hot medium for radial mycelial growth tests in a solid agar medium and (ii) during the incubation phase which was shorter. In addition, the biological reaction was miniaturized in microplate (200 μl for one well against 20,000 μL for one Petri dish): there was less product to use and more space available in chamber growth. Moreover, the number of products to be tested could be greater with more repetitions, and the conditions for calculating the IC_50_ indicator and performing statistical tests were improved. This is one of the reasons why most of the selected EOs showed statistically proven differential activity. The EOs of clove, savory, and thyme showed the best anti-oomycete activities similar to copper sulfate, while oregano, eucalyptus, mint, and tea tree EOs exhibited significantly weaker activities than copper sulfate. For major compounds, three results were obtained: they were statistically more active than their EOs (carvacrol for oregano, D- and L-Carvone for mint) or as active as their EOs sources (thymol for thyme, carvacrol for savory, terpinen-4-ol for tea tree) or less active than their original EOs (eugenol for clove, citronellal for eucalyptus). Carvacrol was the only compound to be as effective as copper sulfate. In order to obtain a better activity than copper sulfate, it would be interesting in the future to study mixtures of EOs and mixtures of major compounds. There are therefore opportunities for EOs and their major compounds to emerge in plant protection to support the reduction in the use of copper.

## Data Availability

Not applicable.
